# Correction: HIF-2α is essential for carotid body development and function

**DOI:** 10.7554/eLife.38781

**Published:** 2018-06-04

**Authors:** David Macias, Andrew S Cowburn, Hortensia Torres-Torrelo, Patricia Ortega-Sáenz, José López-Barneo, Randall Johnson

Macias D, Cowburn AS, Torres-Torrelo H, Ortega-Sáenz P, López-Barneo J, Johnson RS. 2018. HIF-2α is essential for carotid body development and function. *eLife* **7**:e34681. doi: 10.7554/eLife.34681.

Published 19, April 2018

There were several instances in the text, material and methods section and key resources table where the approved mouse gene name was not correctly used.

For example, the title of Figure 1 has been modified as follows:

“Selective loss of carotid body glomus cells in sympathoadrenal-specific *Epas1*, but not *Hif1a*, deficient mice”.

For reference, the original sentence was: “Selective loss of carotid body glomus cells in sympathoadrenal-specific *Hif-2α*, but not *Hif-1α*, deficient mice”.

These have now been corrected.

Owing to a misunderstanding during the proofreading of our article, mouse strains were renamed and italicised in the text and figure legends as *Hif1a^WT^, Th-HIF1a^KO^, Epas1^WT^*, and *Th-Epas1^KO^*. These denominations have been changed back to their previous versions as follows: HIF-1α^WT^, TH-HIF-1α^KO^, HIF-2α^WT^, and TH-HIF-2α^KO^, respectively.

For instance, the legend of Figure 1 has been amended as follows:

“(A and B) Tyrosine hydroxylase (TH) immunostaining on carotid bifurcation sections from HIF-2α^WT^(A, top panels), TH-HIF-2α^KO^ (A, bottom panels), HIF-1α^WT^ (B, top panels) and TH-HIF-1α^KO^ (B, bottom panels) mice (8–12 weeks old).

For reference, the original sentence was:

“(A and B) Tyrosine hydroxylase (TH) immunostaining on carotid bifurcation sections from *Epas1WT*(A, top panels), *Th-Epas1KO* (A, bottom panels), *Hif1aWT* (B, top panels) and *Th-HIF1aKO* (B, bottom panels) mice (8–12 weeks old).”

This error has now been corrected elsewhere to match text and figures.

In addition, the first paragraph of the results section has been amended for clarity as follows:

“To elucidate the role of HIFα isoforms in CB development and function, we generated mouse strains carrying *Hif1a* or *Epas1* embryonic deletions restricted to catecholaminergic tissues (thereafter; TH-HIF-1α^KO^ and TH-HIF-2α^KO^) by crossing them with a mouse strain expressing crerecombinase under the control of the endogenous tyrosine hydroxylase (*Th*) promoter.”

For reference, the original paragraph was:

“To elucidate the role of HIFα isoforms in CB development and function, we generated mouse strains carrying *Hif1a* or *Epas1* embryonic deletions (*Th-HIF1a^KO^* and *Th-Epas1^KO^*) restricted to catecholaminergic tissues by crossing them with a mouse strain expressing crerecombinase under the control of the endogenous tyrosine hydroxylase (*Th*) promoter.”

Owing to a production error there were also several instances where ‘wt’ and ‘ko’ should have been superscripted. These have now been corrected.

Please note that none of these corrections affect the results and conclusions of the original paper.

The article has been corrected accordingly.

